# Proximity of Transmembrane Segments 5 and 8 of the Glutamate Transporter GLT-1 Inferred from Paired Cysteine Mutagenesis

**DOI:** 10.1371/journal.pone.0021288

**Published:** 2011-06-16

**Authors:** Xiuping Zhang, Shaogang Qu

**Affiliations:** 1 China-America Cancer Research Institute, Guangdong Medical College, Dongguan, Guangdong, China; 2 Department of Immunology, Southern Medical University, Guangzhou, Guangdong, China; Ecole Polytechnique Federale de Lausanne, Switzerland

## Abstract

**Background:**

GLT-1 is a glial glutamate transporter which maintains low synaptic concentrations of the excitatory neurotransmitter enabling efficient synaptic transmission. Based on the crystal structure of the bacterial homologue Glt_Ph_, it has been proposed that the reentrant loop HP2, which connects transmembrane domains (TM) 7 and 8, moves to open and close access to the binding pocket from the extracellular medium. However the conformation change between TM5 and TM8 during the transport cycle is not clear yet. We used paired cysteine mutagenesis in conjunction with treatments with Copper(II)(1,10-Phenanthroline)_3_ (CuPh), to verify the predicted proximity of residues located at these structural elements of GLT-1.

**Methodology/Principal Findings:**

To assess the proximity of transmembrane domain (TM) 5 relative to TM8 during transport by the glial glutamate transporter GLT-1/EAAT2, cysteine pairs were introduced at the extracellular ends of these structural elements. A complete inhibition of transport by Copper(II)(1,10-Phenanthroline)_3_ is observed in the double mutants I295C/I463C and G297C/I463C, but not in the corresponding single mutants. Glutamate and potassium, both expected to increase the proportion of inward-facing transporters, significantly protected against the inhibition of transport activity of I295C/I463C and G297C/I463C by CuPh. Transport by the double mutants I295C/I463C and G297C/I463C also was inhibited by Cd^2+^.

**Conclusions/Significance:**

Our results suggest that TM5 (Ile-295, Gly-297) is in close proximity to TM8 (Ile-463) in the mammalian transporter, and that the spatial relationship between these domains is altered during the transport cycle.

## Introduction

Sodium-coupled neurotransmitter transporters are located in the plasma membranes of neurons and glia, where they are present at high density in those areas of the cell membrane that face the synapse. They serve to keep the extracellular neurotransmitter concentrations sufficiently low, so that the postsynaptic receptors are able to detect signaling by the presynaptic nerve cell in the form of exocytotically released transmitters. Thus, neurotransmitter transporters are key elements in the termination of the synaptic actions of neurotransmitters. Moreover, they serve to keep the extracellular transmitter concentrations below neurotoxic levels. Termination of synaptic transmission by transporters takes place with most neurotransmitters, including L-glutamate, *γ*-aminobutyric acid (GABA), glycine, dopamine, serotonin, and norepinephrine.

**Figure 1:**
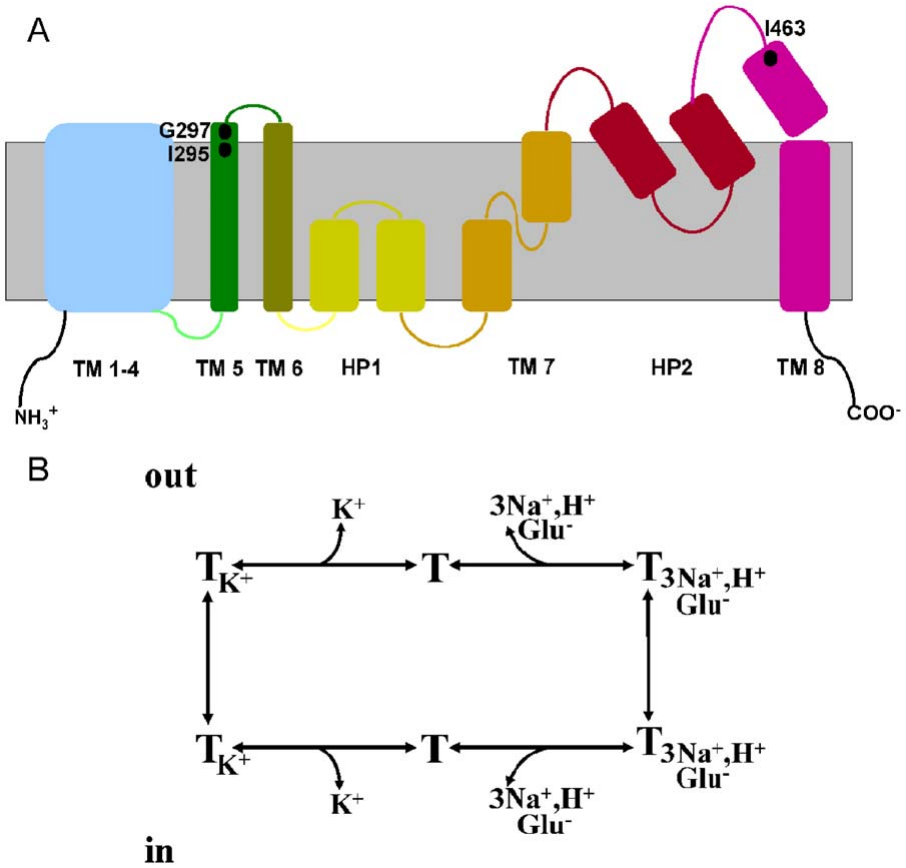
Topology model and transport cycle of GLT-1. (***A***) Topology model of GLT-1 by analogy to that of Glt_Ph_ shown with *black dots* denotes the approximate locations of the following three cysteine substitutions: I295C, G297C, and I463C. TM helices 1– 8 and hairpins (HP1 and HP2) are labeled. (***B***) Transport cycle of GLT-1. After binding of sodium, glutamate, and a proton from the extracellular medium (*up*), the outward-facing substrate-loaded translocation complex is formed. After the external gate closes, the internal gate opens and the substrate and cotransported ions dissociate into the cytoplasm (*bottom*). Subsequently, intracellular potassium enters the binding pocket. After the internal gate closes, the external gate opens and potassium is released into the extracellular medium.

Glutamate transporters have a non-conventional topology (Fig. 1A) containing eight transmembrane segments, two reentrant helical hairpins, first between TM6 and TM7 and the second between TM7 and TM8 [1]–[3]. Moreover, the two reentrant loops are in close proximity [4]. The crystallized Glt_Ph_ transporter has 37% sequence identity with human glial glutamate transporter type one (GLT-l) (also known as excitatory amino acid transporter 2, EAAT2) and the structure was solved at a resolution 3.8 Å [5]. The Glt_Ph_ structure revealed a bowl-shaped structure, formed by a trimer of the transporter, with a solvent-filled extracellular basin extending halfway across the membrane bilayer [5]. At the bottom of the basin three independent binding sites were observed, one in each transporter monomer, suggesting that the monomer is the functional unit. Support for the idea that each monomer functions independently comes from studies with the bacterial glutamate transporter GltT [6] and the neuronal glutamate transporter EAAC1/EAAT3 [7]–[10].

Glutamate transport is an electrogenic process [11]–[13], consisting of two distinct half cycles. First, glutamate is co-transported with three sodium ions and one proton [14], [15] and subsequently the transporter countertransports one potassium ion [16]-[18] (Fig. 1B). Under physiological conditions, the transporter pumps the transmitter into the cell against its concentration gradient [11], [14], [15], but elevated external potassium level causes reverse transport [16], [19]. Thus, in the presence of either high extracellular potassium or L-glutamate, the proportion of transporters in the inward facing conformation will be increased. In this status the binding site is exposed to the cytoplasm. On the other hand, addition of the glutamate's inhibitor, non-transportable glutamate analogues such as D,L-threo-β-benzyloxyaspartate (TBOA) is expected to stabilize an outward-facing conformation of the transporter. In the outward-facing conformation the binding site is exposed to the extracellular medium.

The transmembrane segments TM7 and TM8, together with hairpins HP1 and HP2 have been shown to enclose non protein density which presumbly correspond to glutamate [5]. The Glt_Ph_ structure represents a static picture of a substrate-occluded conformation of the transporter [5]. The TBOA-bound structure [20], where the proposed extracellular gate, HP2, has moved toward the extracellular space, resembles the outward-facing conformation of the transporter. However, during a translocation cycle, the transporter transits through many other conformations. To assess the proximity and functional significance of residues in TM5 and TM8 of the cysteine-less version of GLT-1 (CL-GLT-1, in which the endogenous cysteines were replaced by serine, so that the interaction between the induced and endogenous cysteines is abolished), we engineered pairs of cysteine residues (I295C/I463C and G297C/I463C) into TM5, TM8 and examined the impact of disulfide cross-linking with Copper(II)(1,10-Phenanthroline)_3_ on transport activity (Fig. 1A). Such cross-linking often results in the inhibition of transport [4], [21], [22]. The inhibition may be due to restrictions imposed by the disulfide cross-link on the conformational changes, which the transporter undergoes during a transport cycle or may be the result of a steric barrier or another distortion introduced by the crosslink. In this study, we have used two types of functional assays to infer proximity of engineered cysteine pairs. The double mutants were subjected to conditions of oxidative cross-linking in the presence and absence of transporter ligands. We report here the identification of two cysteine pairs, I295C/I463C and G297C/I463C, which behave as if they are close together. The data provides evidence that TM5 and TM8 are spatially close to one another, and that the spatial relationship between these domains is altered during the transport cycle.

## Results

### Effects of thiol cross-linking and Cd^2+^ on transport

**Figure 2:**
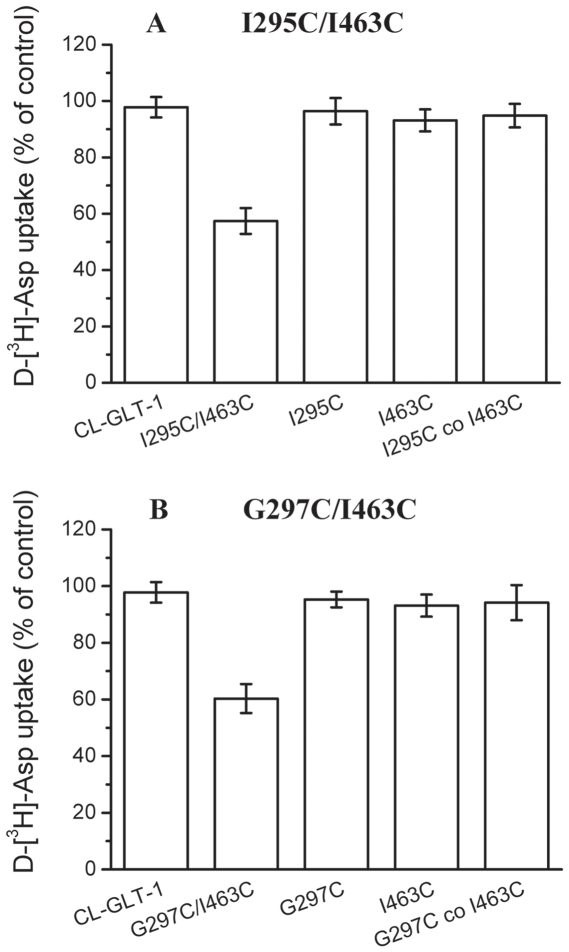
Effect of CuPh on the activity of cysteine mutants. HeLa cells expressing double cysteine mutants or the indicated control mutants, all in the background of CL-GLT-1, were preincubated in NaCl-containing medium with 200 µM CuPh for 5 min at room temperature, washed twice with choline chloride-containing solution, and subsequently D-[^3^H]aspartate transport was assayed. Co-expression of two single cysteine mutants in HeLa cells is marked by “co”. The values shown represent the percentage of activity after treatment with 200 µM CuPh relative to that obtained after preincubation in the absence of CuPh. Values represent the mean ± S.E. of at least three separate experiments each done in triplicate. (***A***) I295C/I463C double cysteine mutants and its control mutants. (***B***) G297C/I463C double cysteine mutants and its control mutants.

To identify positions in TM5 and TM8, which are potentially close to each other, we constructed 11 double cysteine transporters for this cross-linking study. To determine whether the cysteine pair introduced into each transporter is capable of forming a disulfide bond, we expressed each transporter in HeLa cells and then measured the accumulation of radiolabeled D-aspartate before and after exposure to the cross-linking reagent CuPh. From this assay, we identified two double cysteine transporters, I295CC/I463C and G297C/I463C (Fig. 2A and B), that exhibit a dramatic decrease in transport activity following exposure to CuPh. The other transporter mutants showed either impaired transport activity in the absence of 200 µM CuPh (L294C/L465C, L294C/L466C, I295C/L466C) or no change in transport activity after exposure to CuPh (L294C/I463C, I295C/E461C, I295C/L465C, G297C/L465C, G297C/L466C, K298C/E461C) (data not shown).

When HeLa cells, expressing I295C/I463C or G297C/I463C, were preincubated with the oxidizing agent CuPh (200 µM), a significant inhibition of transport was observed (Fig. 2A and B). This inhibition was not seen with cells expressing either CL-GLT-1 or the single cysteine mutants I295C, G297C and I463C (Fig. 2A and B), indicating that the inhibition of transport by oxidative cross-linking required a cysteine at both positions. The inhibition by CuPh was only observed when the cysteines at positions 295 and 463 or 297 and 463 were present on the same polypeptide, but not when the two cysteines resided on two different polypeptides. This was demonstrated by the lack of inhibition by CuPh of transport in cells cotransfected with I295C and I463C or G297C and I463C (Fig. 2A and B). This suggests that the cysteines at positions 295 and 463 or 297 and 463 come into close proximity within the transporter monomer, but not at the interface of the two transporter monomers. To better characterize the effect of CuPh on the I295C/I463C and G297C/I463C transporters, we measured D-[^3^H] aspartate transport activity as a function of CuPh concentration. For both transporters, we observed that increasing concentrations of the cross-linking agent (10–600 µM) lead to a greater reduction in D-aspartate transport (data not shown). At the 600 µM of CuPh, the transport activity was almost abolished. The inhibition of transport of I295C/I463C and G297C/I463C by CuPh could be reversed by a subsequent incubation with 20 mM dithiothreitol (DTT) (data not shown). In rare instances CuPh can lead to the formation of covalent links between cysteine and other residues and thus the reversibility in the presence of DTT confirms the formation of a disulfide bond.

**Figure 3:**
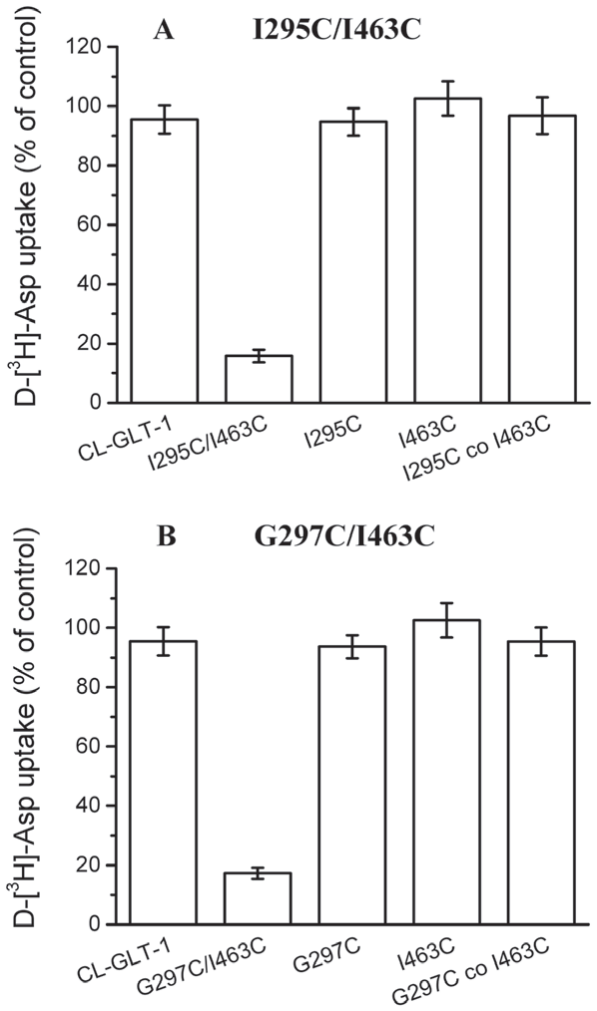
Inhibition of transport of cysteine mutants by Cd^2**+**^. HeLa cells expressing the indicated mutants were washed once with choline chloride-containing solution and assayed for transport in the presence or absence of 500 µM cadmium chloride. Values shown are the percentage activity in the presence of 500 µM cadmium chloride relative to that in its absence. Values represent the mean ± S.E. of at least three separate experiments each done in triplicate. (***A***) I295C/I463C double cysteine mutants and its control mutants. (***B***) G297C/I463C double cysteine mutants and its control mutants.

Although the strongest inhibition of transport by CuPh was observed in the I295C/I463C and G297C/I463C double mutants, we looked for additional evidence that these two positions could be close in space and examined the ability of the I295C/I463C and G297C/I463C double mutants to form a high affinity Cd^2+^ binding site. This divalent cation interacts with cysteinyl side chains [23], [24], and the affinity of the interaction is dramatically increased when the Cd^2+^ can be coordinated by two cysteines [25]. Exposure of the single mutants I295C, G297C and I463C to up to 500 µM Cd^2+^ had very little effect on D-[^3^H] aspartate uptake (Fig. 3A and B). In contrast to these controls, an inhibition of ∼85% is observed on uptake by the I295C/I463C and G297C/I463C mutants (Fig. 3A and B). The inhibition by Cd^2+^ was only observed when the cysteine pairs were introduced in the same polypeptide (Fig. 3A and B) but not when the single mutants were coexpressed. This suggests that the cysteines introduced at positions 295 and 463 or 297 and 463 come in close proximity within the transporter monomer but not at the interface of two transporter monomers. Our observations from cross-linking and the effects of Cadmium Ions thus far suggest that Ile-295 and Gly-297 in TM5 is indeed in close proximity to Ile-463 in TM8.

### Effect of Glutamate and TBOA on cross-linking in double cysteine transporters

**Figure 4:**
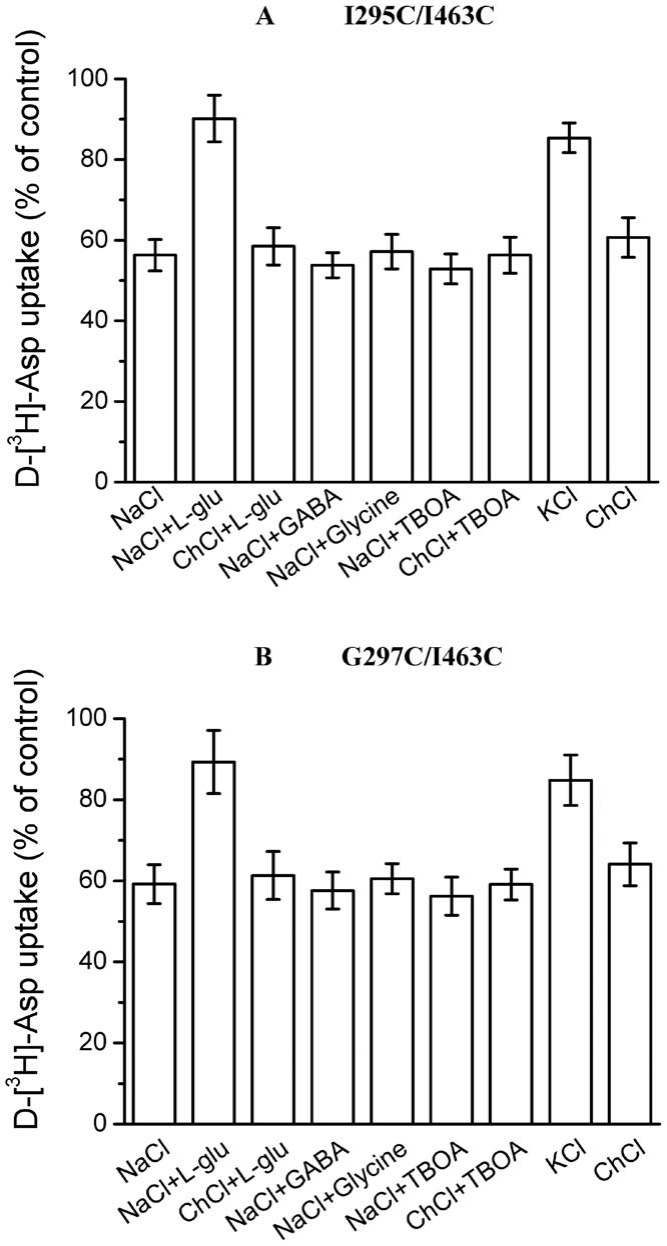
Effect of the composition of the external medium on the inhibition of double cysteine mutants by CuPh. HeLa cells expressing double cysteine mutants were preincubated for 5 min in the presence and absence of 200 µM CuPh. The indicated preincubation solutions contained NaCl, NaCl +1 mM L-glutamate, ChCl +1 mM L-glutamate, NaCl +1 mM GABA, NaCl +1 mM glycine, NaCl +20 µM TBOA, KCl, choline chloride. Values are given as percent of control (preincubation without CuPh) and represent the mean ± S.E. of at least three different experiments done in triplicate. (***A***) I295C/I463C double cysteine mutants. (***B***) G297C/I463C double cysteine mutants.

The reaction with CuPh and cysteines results in the formation of a covalent bond, so it is possible to determine the effect of the external medium on the cross-linking during the pretreatment of the cells with CuPh. When during pretreatment of cells expressing I295C/I463C and G297C/I463C sodium was replaced by choline, there was not much change in the extent of inhibition by CuPh (Fig. 4A and B). When the sodium-containing medium was either supplemented with glutamate or replaced by potassium, conditions that promote the formation of the inward-facing conformation, a marked reduction in the degree of inhibition by CuPh was observed (Fig. 4A and B). This suggests that the cysteine residues are far apart in the inward-facing conformation. The protection by L-glutamate was not seen in the absence of sodium (choline replacement; Fig. 4A and B) and was not observed with GABA or glycine, which are not substrates of GLT-1 (Fig. 4A and B). The non-transportable substrate analogue TBOA is expected to increase the proportion of outward-facing transporters, while it had no significant effect on this inhibition (Fig. 4A and B).

**Figure 5:**
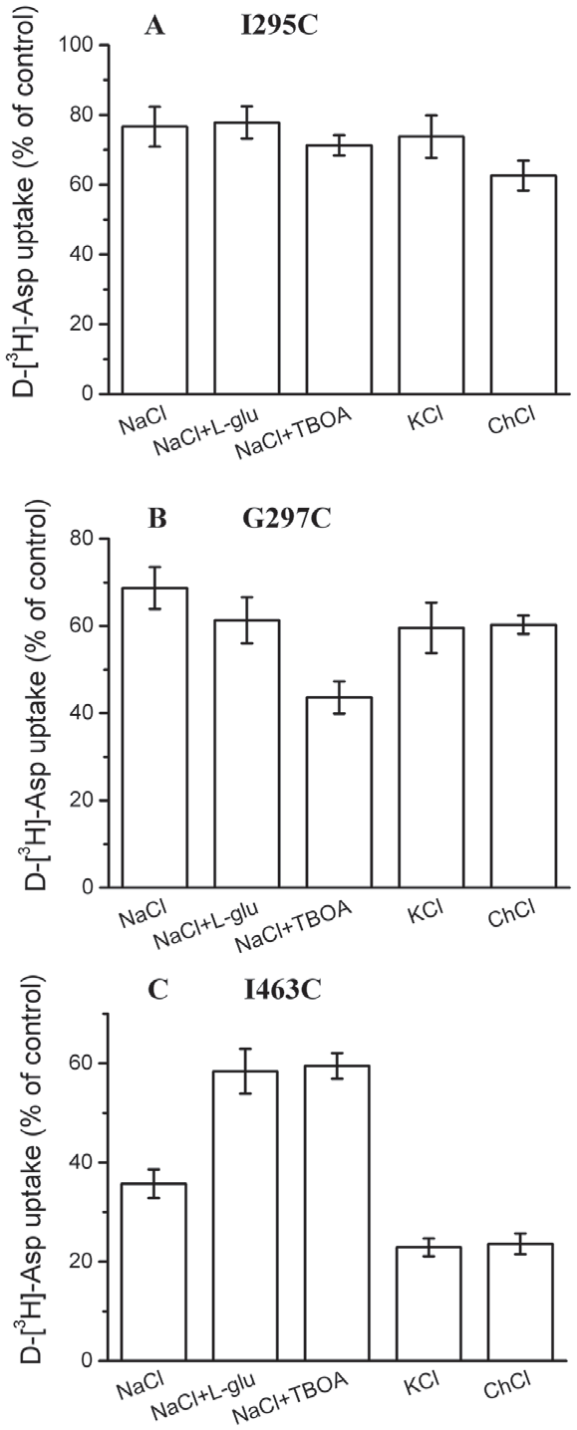
Effect of the composition of the external medium on the inhibition of single cysteine mutants by MTSET. Cells expressing the single cysteine mutants I295C (***A***), G297C (***B***) or I463C (***C***), were preincubated for 5 min in the presence or absence of 1.0 (***A***), 0.6 (***B***) or 0.03 (***C***) mM MTSET. The indicated preincubation solutions contained NaCl, NaCl +1 mM L-glutamate, NaCl +20 µM TBOA, KCl, choline chloride. Values are given as percent of control (preincubation without MTSET) and represent the mean ± S.E. of at least three different experiments done in triplicate.

In principle, the modulation of the inhibition by CuPh could be a result of changes in accessibility of the engineered cysteine residues, rather than in their distance. As a measure of their aqueous accessibility, we determined the effect of MTS reagents on transport by the single cysteine mutants. Preincubation of I295C with the membrane-impermeable sulfhydryl reagent MTSET [(2-trimethylammonium) methanethiosulfonate] resulted in inhibition of transport. Glutamate and external potassium, which protected against cross-linking of the cysteine pairs (Fig. 4A), did not modulate the inhibition of I295C by MTSET, and this was also true for TBOA (Fig. 5A). Preincubation of G297C with MTSET also resulted in inhibition of transport, which was potentiated by TBOA (Fig. 5B). However, Glutamate and external potassium, which protected against cross-linking of the cysteine pairs (Fig. 4B), did not modulate the inhibition of G297C by MTSET (Fig. 5B). Previously, L-glutamate and TBOA were also shown to protect against the inhibition of transport of I463C by MTSET [26]. With the higher concentration of MTSET, a similar protective effect was also observed with glutamate and TBOA (Fig. 5C), which again is different from the cross-linking results. Thus, while the accessibility of the introduced cysteines to MTSET appears to be dependent on the conformational state of the transporter, the effects of substrates and substrate analogues on cross-linking cannot be explained merely in terms of such changes in accessibility.

## Discussion

Glutamate transporters play an important role in the uptake of the neurotransmitter. The study of glutamate transporters has extremely important significance for the medical field. Glutamate possesses a dual function. As the main internal excitory neurotransmitter, it is also a potential endogenous neurotoxin. Under normal biological conditions, the glutamate transporters, which are located on neurons and glial cells, rapidly uptake glutamate, effectively decreasing glutamate accumulation in the synapse. While in a certain pathological environment, if the glutamate transporter's activity decreases, or if the direction of glutamate transporter uptake is reversed, the result will be that the concentration of glutamate will increase in the synapse, and glutamate will excite glutamate receptors and trigger a wave of excitotoxicity. Glutamate transporters are one of the subjects under investigation for the treatment of degenerative diseases of the central nervous system. Consequently, by making progress in the study of glutamate transporters, the mechanism of the degenerative diseases can be better understood, and we will be able to find some clues for the treatment of degenerative diseases of the central nervous system.

From our experiments it was discovered that CuPh and Cd^2+^ could inhibit the transport activity of the I295C/I463C and G297C/I463C double cysteine mutants (Figs. 2 and 3). This can be explained by what was observed during the transport. The positions of Ile-295, Gly-297 and Ile-463 became so close, that in the presence of CuPh, a disulfide bond was formed between I295C and I463C as well as between G297C and I463C. Once the disulfide bond was formed, the structure of the transporter was locked and couldn't change anymore. The other outcome was the two cysteines, closely positioned to each other, interacted with Cd^2+^, which also led to the locking of the transporter's structure. During the transport the transporter's molecular structure is constantly undergoing change [27], if the structure is locked; the activity of transporter will be severely inhibited.

During the substrate uptake process, the structure of the transporter is changing constantly, thus resulting in the distances among the different segments also constantly changing. If substrates or potassium are added to the outside of cell, the transporter will open up to the cytoplasm. If the substrate's inhibitor is added to the outside of the cell, the transporter will open up to the outside of the cell. We performed different tests with substrates, potassium, and the substrate's inhibitor for their impact on the CuPh inhibition effect. From these experiments we tried to determine the different distances between Ile-294 and Ile-463 as well as Gly-297 and Ile-463 during different transport phases. Substrates, potassium, and the substrate's inhibitor may also have an impact on the inhibition by impermeant sulfhydryl reagent MTSET of the single cysteine mutant. We also tested this type of impact in order to explore the single cysteine mutants' accessibility during the transport substrate process. For the I295C/I463C and G297C/I463C double cysteine mutants, comparing with sodium, glutamate and potassium had protective effect on the inhibition by CuPh (Fig. 4A and B). TBOA had no significant effect on this inhibition (Fig. 4A and B). While comparing to the effects on the cross-linking, TBOA and glutamate had different effects on the inhibition of transport of single cysteine mutants by MTSET. TBOA increased the inhibition of transport of G297C by MTSET (Fig. 5B), and decreased the inhibition of transport of I463C by MTSET (Fig. 5C). Glutamate has no effect on the inhibition of transport of I295C and G297C by MTSET (Fig. 5A and B). From these results we can conclude that in addition to an effect on accessibility, glutamate can cause a relative movement between TM5 and TM8. Because the trimeric interface involving TMs 2, 4, and 5 is known to be unchanged during transport [6], we assume that this conformational change would most likely involve TM8. In the inward-facing conformation of the glutamate transporters, after binding with the substrate, the protein core consisting of HP1, TM7, HP2, and TM8 moves inward relative to the rest of the protein to form a cytoplasmfacing conformation [28]. On the other hand, TM8 also moves back so that the 295, 297 and 463 positions get far away. The findings of these studies confirm that TM5 (Ile-295, Gly-297) is in close proximity to TM8 (Ile-463) in the mammalian transporter, and that these residues are repositioned with respect to each other at different steps in the transport cycle.

The observation that position 295 and 297 at the end of TM5 is close to position 463 (Figs. 2, 3, and 4), located at the top of TM8, enables us to refine the topological model of GLT-1. Proximity of transmembrane segments 5 and 8 of the glutamate transporter GLT-1 is different from the situation in Glt_Ph_, where at these pairs positions the distance are >20 Å apart in the crystal structures of Glt_Ph_ [5]. The two transporters are different in this regard. Comparing Glt_Ph_, the eukaryotic glutamate transporters have an additional extracellular domain, which contains the *N*-linked glycosylation sites. Obviously, its structure and its relationship with the rest of the transporter are as yet unknown.

The substrate analogue TBOA, expected to cause an increase of the proportion of outward-facing transporters, increased the inhibition by MTSET in TM5 mutants with

cysteine introduced at position 297 (Fig. 5B). In the TBOA-bound Glt_Ph_ structure, HP2 has moved toward the extracellular side, away from the binding pocket [20]. Some other part of the transporter has moved together with HP2. All these changes lead to the increase of the accessibility of Gly-297 (Fig. 5B).

## Materials and Methods

### Generation and Subcloning of Mutants

The CL-GLT-1 in the vector pBluescript SK(–) (Stratagene) will be used as a parent for site-directed mutagenesis as described previously [29], [30]. Briefly the parent DNA was used to transform Escherichia coli CJ236 (dut–, ung–). From one of the transformants, single-stranded uracil-containing DNA was isolated upon growth in uridine-containing medium according to the standard protocol from Stratagene using helper phage R408. This yields the sense strand, and consequently mutagenic primers were designed to be antisense. The mutants were subcloned into constructs containing CL-GLT-1 in the vector pBluescript SK(–), using the unique restriction enzymes EcoRI and BsrGI or BsgI and XbaI. The coding and non-coding strands were sequenced between the above restriction sites.

### Cell Growth and Expression

HeLa cancer cell line was purchased from ATCC (Manassas, VA). HeLa cells were cultured in Dulbecco's modified Eagle's medium (DMEM) supplemented with 10% fetal calf serum (FCS), 200 units/ml penicillin, 200 µg/ml streptomycin, and 2 mM glutamine. Heterologous expression of the wild type and mutant transporters was done as follows: HeLa cells plated on 24-well plates were infected with recombinant vaccinia/T7 virus vTF [31] by application of 150 µL of the virus/DMEM mix (lacking FCS) and incubation at 37°C for approximately 30 min prior to transfection with DNA (pBluescript SK with the wild type or mutant transporter inserted downstream to the T7 promoter) using the transfection reagent DOTAP. Transfection was carried out by applying 200 µL of the DNA/DOTAP/DMEM mix (lacking FCS) as described [32]. Cells were incubated at 37°C until transport assay.

### Transport

Uptake of D-[^3^H]-aspartate into whole cells was assayed 18–20 h post transfection. The wells were washed twice with the choline solution (150 mM choline chloride, 5 mM KP_i_, pH 7.4, 0.5 mM MgSO_4_, and 0.3 mM CaCl_2_). Each well was then incubated with 200 µL transport medium (150 mM NaCl, 5 mM KP_i_, pH 7.4, 0.5 mM MgSO_4_, and 0.3 mM CaCl_2_) supplemented with 0.4 µCi of the tritium-labeled substrates and incubated for 10 min at room temperature, followed by washing, solubilization of the cells with SDS, and scintillation counting.

### Inhibition Studies with Sulfhydryl Reagents

Before the transport measurements, the cells adhering to 24-well plates were washed with the choline solution. Each well was then incubated at room temperature with 200 µL of the preincubation medium (the different compositions are indicated in the figure legends). After 5 min, the medium was aspirated, and the cells were washed twice with 1 ml of the choline solution. Subsequently, they were assayed for D-[^3^H]-aspartate transport at room temperature. Each experiment was performed at least three times. MTSET were purchased from Anatrace, Inc. The concentration of MTSET chosen in the different experiments was optimized according to the mutants used.

### Inhibition of transport by Copper(II)(1,10-Phenanthroline)_3_

HeLa cells transfected with the indicated constructs were washed once with choline solution and preincubated with the indicated concentration of Copper(II)(1,10-Phenanthroline)_3_ (CuPh). After 5 min, the medium was aspirated, and the cells were washed twice with 1 ml of the choline solution followed by the transport assay using 200 µL transport medium supplemented with 0.4 µCi of the radiolabeled amino acid for each well. Each experiment was performed at least three times. Again, the optimal concentration of CuPh for each double mutant was determined by preliminary titration experiments. The CuPh stock solution was prepared for each experiment by mixing 0.4 ml of 1.25 M 1,10-phenanthroline in water:ethanol (1:1) and 0.6 ml of 250 mM CuSO_4_.

### Inhibition of transport by Cd^2+^

HeLa cells transfected with the indicated construct were washed once with choline solution and preincubated with the indicated concentrations of cadmium chloride in transport solution (150 mM NaCl, 5 mM KP_i_, pH 7.4, 0.5 mM MgSO_4_, and 0.3 mM CaCl_2_) with radiolabelled D-aspartic acid for 10 min at room temperature.
